# Recognition criteria for occupational cancers in relation to hepatitis B virus and hepatitis C virus in Korea

**DOI:** 10.1186/s40557-018-0217-0

**Published:** 2018-01-31

**Authors:** Hogil Kim, Yun Kyung Chung, Inah Kim

**Affiliations:** 1 Department of Occupational and Environmental Medicine, Hanyang University College of Medicine, 222-1, Wansimniro, Seongdonggu, Seoul, 04763 South Korea; 2 Korea Medical Institute, Seoul, South Korea

**Keywords:** Cancer, Occupation, Hepatitis B virus, Hepatitis C virus

## Abstract

The goal of this study was to review the scientific basis for the recognition of occupational cancer, in relation to hepatitis viral infections in Korea. Most Hepatitis B virus (HBV) infections in Korea occur as vertical infections, but these are decreasing rapidly due to vaccination. Hepatitis C virus (HCV) is known to be transmitted through parenteral routes, but the transmission route is often unclear. Most occupational infections of hepatitis virus involve accidental injuries of medical institution workers while using virus-contaminated medical devices. Many cohort studies and case-control studies have consistently reported that HBV and HCV infection increases the risk of hepatocellular carcinoma (HCC) and the strength of this association is high. Non-Hodgkin’s lymphoma appears to be associated with HCV. Cholangiocarcinoma, pancreatic cancer, leukemia, and thyroid cancer are considered to be less related or unrelated to epidemiological causation. There are no uniform international specific criteria for occupational cancer caused through occupational exposure to a hepatitis virus. In establishing appropriate standards applicable to Korea, there should be sufficient consideration of latency, virus exposure levels and frequency, and other cancers, apart from HCC.

In conclusion, we recommend keeping the current specific criteria. However, if a worker is injured at work when using a sharp medical device, and HBV and HCV viral infections are confirmed through serologic tests; if the worker is diagnosed as having a chronic HBV or HCV infection, a subsequent HCC (or Non-Hodgkin’s lymphoma following chronic HCV infection) can then be considered highly related to the worker’s occupation.

## Background

Globally, chronic hepatitis B and hepatitis C are the most common causes of liver cancer [1, 2]. Korea is no exception, and Korean National Cancer Registry data indicate that 74.2%, 8.6%, and 6.9% of liver cancers are caused by Hepatitis B, Hepatitis C and long-term excessive alcohol consumption, respectively [3]. In 1994, the International Agency for Research on Cancer (IARC) approved Group I classification for the carcinogenicity of the hepatitis B virus (HBV) and hepatitis C virus (HCV) [4].

Infection by the hepatitis virus is one of the most common diseases among occupational infections [5]. Annually, health care workers experience between 600,000 and 800,000 exposures to blood in the US [6], with approximately 40% of HBV and HCV cases in these individuals resulting due to occupational exposure [7]. In Korea, analysis of 10,619 occupational diseases that were compensated for nine years from 1997 to 2007 by the Korea Occupational Safety and Health Agency, showed that 14% of the 851 cases involved viral hepatitis [8]. Another study analyzing the infectious diseases recognized as work-related diseases by the Industrial Accident Compensation Insurance (ICAI) Act from 2006 to 2011, found that 1062 cases of these cases involved employees of medical institution, and viral hepatitis accounted for 5.2% of these cases [9].

Viral hepatitis due to occupational exposure is a prerequisite for occupational liver cancer caused by HBV and HCV. However, there is no evidence that hepatitis caused by hepatitis virus has been recognized as an occupational disease in Korea. As a result of reviewing all cases in which we filed for occupational diseases at the Korea Labor Welfare Corporation from 2010 to 2016, there were a few cases where liver cancer was recognized as an occupational disease. None of the applicants for health care workers were found to have a high probability of occupational infection. In the epidemiological survey of the Institute of Occupational Safety and Health, no virus caused by virus was found. Many healthcare workers in Korea are not covered by industrial accidents insurance, but are members of Government Employees Pension Service or Teachers’ pension. In consideration of this, we investigated cases of occupational disease compensation announced by two special occupational pension corporations, but it was impossible to confirm it because it was disclosed only in terms of the number of incidents.

Although no case of hepatocellular carcinoma due to occupational exposure has been applied or recognized as an occupational disease, it is inevitable that the situation changes because viral hepatitis due to occupational exposure is recognized. However studies on occupational cancers (including liver and other cancers) examining exposure routes, levels, and latency periods caused by occupational hepatitis virus infection in Korea are limited. Moreover, no consensus was achieved for the recognition of occupational cancer based on these results. Therefore, the aim of this study was to conduct a review, focusing on the scientific basis for cancer caused by occupational hepatitis virus infection, the status of exposure in Korea, and the current international specific criteria. Furthermore, this information could be used as a reference when applying occupational cancer standards by occupational environmental medicine experts for better decisions-making.

### Chronic viral hepatitis

Hepatitis is a disease that causes inflammation of hepatocyte tissue, and is often caused by viral infections. Viral hepatitis is classified as acute or chronic, according to its natural history. Usually, patients with acute hepatitis recover within 3–4 months after onset, but chronic hepatitis lasts more than 6 months and for several years. The types of viruses causing chronic viral hepatitis include types B, C, D, and G. Almost all cases of hepatitis (99%) in Korea are either type B or C [10].

Diagnosis of a chronic HBV infection is possible when liver damage can be detected following disease onset. Serologically with HBV infection, hepatitis B surface antigen (HBsAg) and the hepatitis B e-antigen (HBeAg) are present, HBV DNA is moderate to severe, and serum alanine aminotransferase (ALT) levels are increased [11–13]. In addition, an anti-HBe antibody, which means that virus replication is activated, appears and is known as HBeAg-positive chronic hepatitis B infection. In some cases, viral replication progresses to an HBeAg-negative state, which is referred to as HBeAg-negative chronic hepatitis B. An inactive carrier patient is defined as: HBsAg is present, HBeAg is absent, the anti-HBe antibody is present, the HBV DNA level is low and the ALT level is normal. In most patients at this stage, the infection has improved and less than 5% of the cases are known to progress to a chronic infection state without loss of infection [13, 14]. The chances of progressing to become chronic carriers vary according to the time of infection with HBV, which involves a 90% probability in infected newborns, 50% in infancy, 20% in childhood and 5% in adults [15–17]. Between 25% and 40% of HBV carriers progress to cirrhosis via hepatitis, and hepatocellular carcinoma (HCC) occurs in 1% to 5% of liver cirrhosis cases annually [13].

Unlike HBV, HCV infection progresses to chronic infections regardless of the route or timing of infection [18, 19]. Clinically, it is difficult to distinguish between acute and chronic hepatitis due to HCV infection. However, since almost all infections progress to chronic hepatitis [20, 21], serologically confirmed cases of hepatitis C virus infection, such as the presence of anti-HCV antibody and HCV DNA positive, can be regarded as chronic infections. HCV infection also has a similar prognosis as HBV. Approximately 70% to 80% of infected individuals progress to chronic disease, and then approximately 20% of these progress to cirrhosis and approximately 5% to 10% develop liver cancer [22].

### Source of exposure in Korea

The pathway of HBV infection is parenteral, including infection from a mother who is an HBV carrier, sexual contact, contact with infected blood, tattooing, sharing of needles between drug users, and most result from vertical infection [13, 23]. However, the prevalence is decreasing because of vaccinations to prevent vertical infection. In Korea, after the introduction of the HBV vaccine in 1983, the HBsAg-positive rate of 12% in mothers steadily decreased to 4.5% in 1998 and to 3.7% in 2005, and has remained at a similar level since then [24]. HCV infection is also primarily parenteral, with transmission through sexual intercourse or blood [20]. In Korea, HCV testing for donated blood was only introduced after 1992. Because of this, some people who have had blood transfusions prior to this date have been infected and have been diagnosed with chronic hepatitis C infection [25]. Vertical infections are reported to be less than 5%, which is very low compared to HBV [26]. In addition, there are many HCV patients who do not know when they were exposed to HCV, and these may be examples of community-acquired infections [27].

Occupational exposure to hepatitis viruses most often results from injury due to sharp-edged medical devices contaminated with virus-containing blood or injectable preparations. In Korea, the Institute of Occupational Safety and Health has reported a total of 9.4 cases per 100 hospital beds since 2009 [28]. However, the report rate of needle-stick injuries was very low [29], and in reality was estimated to be much higher. Needle-stick injuries can theoretically occur in any person working in a medical institution. Both in the US and Korea, it has been reported that injuries occur most commonly among nurses (including assistants) [8]. The most common medical devices causing transdermal injuries were reported to involve syringes (70%) and stitches (10). Since HBV is resistant to drying, room temperature, and alcohol disinfection, it can survive for up to one week in a normal environment [30]. Thus, when dealing with medical devices, workers need to be careful because these could be potentially infectious.

The probability of infection with a hepatitis virus when exposed to a single needle-stick injury is also an important concern. It is known that the likelihood of exposure to HBV following a single needle-stick injury is between 3% and 10%, and if the person is HBV e- antigen positive, it is between 22 and 31% higher [22, 31]. In the case of HCV, the probability of conversion to HCV-positive during transdermal exposure to blood was 3% [22]. Infections after mucosal exposure have been reported to be lower than the transfusion rate, and some cases have been reported [32].

### Epidemiologic study of human carcinogenicity

#### Hepatocellular carcinoma (HCC)

As noted, the IARC classified HBV and HCV as Group 1 in 2011, because they are biological risk factors with sufficient evidence for causing HCC in humans. In the US, the National Toxicology Program classified HBV as "known to be a human carcinogen" in 2003, following the same classification for HCV in 2002. The American Conference of Governmental Industrial Hygienists (ACGIH) assigned HBV an A2 designation as a suspected human carcinogen in 2007.

The IARC reviewed 12 cohort studies of hepatocellular carcinoma and HBV infection (Table 1). Seven studies were conducted in Asia [33–39], 2 in Europe [40, 41] and 1 in Africa [42], the US [43] and Australia [44]. There was no difference in the risk of hepatocellular carcinoma according to sex. The follow-up period ranged from 4 years [44] to 22 years [40]. Most studies used an HBsAg serum-negative group as the control group, but some studies used the general population as the control group [40, 44]. The results of most cohort studies suggest that the relative risk of chronic HBV infection and HCC is consistently high even when adjusted for anti-HCV antibody, smoking, alcohol consumption and blood glucose levels. The magnitude of the relative risk varied from 9.6 (95% CI 6.0–15.2) [35] to 74 (95% CI 45–121) [38].

**Table 1:** Cohort studies of HBV surface antigen (HBsAg)-seropositive people for the development of HCC

Authors(Years), Country	Cohort description	No. of cases of HCC	Result RR(95% CI)
Evans et al. (1998) [42] Senegal	Men in Senegalese Army, 2611 HBsAg+ / 10,425 HBsAg-	14	NR
Nomura et al. (1996) [43] Hawaii, USA	Men of Japanese ancestry (born 1900–1919), 7498 HBsAg+	28	43.0 (5.7–325.5)
Chang et al. (1994) [33] Taiwan, China	Men (30–85 years), NR HBsAg+ 38 HCC(24HBsAg+) 152 controls (10 HBsAg +)	24 HBsAg+ 14 HBsAg-	26.48 (7.92–88.56) Excluding HCV+ 44.57 (10.17–195.30)
Lu et al. (1998) [34] Qidong, China	Men (20–60 years), 737 HBsAg+ 699 HBsAg-	47	11.61
Yang et al. (2002) [35] Taiwan, China	Men (30–65 years), 2361 HBsAg+ (1991 HBsAg+/HBeAg-) (370 HBeAg+/ HBeAg+) 9532 HBsAg-/HBeAg-	82 50 32 29	[11.51 (7.54–17.57)] 9.6 (6.0–15.2), 60.2 (35.5–102.1) 1.0
Evans et al. (2002) [36] Haimen City, China	Men and women (25–64 years), Men 8795 HBsAg+ 49,659 HBsAg- Women 2711 HBsAg+ 22,629 HBsAg-	643,257 61 16	18.8 (16.0–22.1) Adjusted for other factors 33.2 (17.0–65.0) Adjusted for other factors
Wang et al. (2003) [37] Taiwan, China	Men (30–65 years), 2416 HBsAg+ 9421 HBsAg-		13.1 (8.6–20.1)
Tanaka et al. (2004) [38] Osaka, Japan	Blood donors (men and women 40–64 years), 2519 HBsAg+/ HCV- 1927 HCV+/ HBsAg- 25 HCV+ /HBsAg+ 150,379 HCV−/ HBsAg-		74 (45–121) 36 (21–62) 161 (46–557) 1.0 Adjusted for sex, age, ALT level and cholesterol level.
Gwack et al. (2007) [39] Korea	Men and women 30 +, HBsAg+ 296 HBsAg- 6582	10 26	[9.6 (4.7–19.9)]
Amin et al. (2006) [44] Australia	People notified as HBV or HCV positive to NSW State Health Department, All 39,109 HBsAg 75,834 HCV 2604 HBV/HCV Men 20,808 HBsAg 47,903 HCV 1932 HBV/HCV Women 18,301 HBsAg 27,931 HCV 672 HBV/HCV	124,143 6 105,101 5 19 42 1	30.6 (25.7–36.5) 22.5 (19.1–26.5) 30.3 (13.6–67.5 30.6 (25.3–37.1) 18.7 (15.4–22.7) 27.5 (11.4–65.9) 30.7 (19.6–48.1) 44.0 (32.6–59.6) 64.2 (9.0–455.8)
Crook et al. (2003) [40] England and Wales	Blood donors, 2681 HBsAg+ men 977 HBsAg+ women National population rates as comparator	20 1	26 (16.0–40.5) 10 (NR)
Ribes et al. (2006) [41] Spain	Blood donors, 1656 HBsAg+ men 631 HBsAg+ women 8783 HBsAg-men 6721 HBsAg-women	14 1 NR NR	14.1 (7.7–23.6) 1.5 (0.02–8.29)

HCC, Hepatocellular carcinoma; RR, Relative risk

The IARC also reviewed 31 case-control studies in the same report, 14 of which were conducted in Asia [45–58], 9 in Europe [59–67], 7 in Africa [68–74] and 1 in the United States [75]. When the HBsAg level was positive, the adjusted odds ratios of HCC ranged from 1.5 to 87.4. In some studies, combined infection with HCV has been shown to act as an independent risk factor after adjusting for alcohol consumption, smoking, and diabetes [46, 48, 49, 51, 52, 62, 67].

Eight cohort studies on HCV infection and HCC are reviewed in Table 2. In 6 studies conducted in Asia [33, 37, 76–79], one in the United States [80] and one in Australia [44], the relative risk varied from 2.5 to 88. These wide ranges of relative risk arise from differences in the duration and prevalence of HCV infection. Nonetheless, except for one cohort study [76], all of these reported statistically significant results. HBV infection, a potential confounding factor, was only reviewed in 4 of these studies. There was one study that included differences in the titers of anti-HCV antibodies, with a higher titer reported to have a stronger association with HCC [78].

**Table 2:** Cohort studies of HCV and HCC

Authors(Years), Country	Cohort description	Detection method, No. of cases of HCC	Result RR(95% CI)
Guiltinan et al. (2008) [80] USA	10,259 anti-HCV-positive allogeneic blood donors (6627 men, 3632 women) and 10,259 anti-HCV-negative donors (6627 men, 3632 women), Average follow-up: 7.7 years	Anti-HCV, Neg 1 Pos 16	1.0 16.6 (2.2–125.5), Matched on sex, age, and zip code
Chang et al. (1994) [33] Taiwan, China	9775 men (aged 30–85 years), enrolled between 1984 and 1986 and followed to 1992 (years of follow-up: 0.5–6.0 years)	Anti-HCV, Neg 33 Pos 5	1.0 88.2 (5.2–1509) Adjusted age, residence, date of recruitment, HBsAg, vegetable consumption, and chronic liver disease history
Yuan et al. (1995) [76] Shanghai, China	18,244 men (aged 45–64 years), enrolled between 1986 and 1989 and followed to 1993	Anti-HCV, Neg 75 Pos 1	1.0 5.0 (0.3–79.9), Adjusted age, time of sample collection, and neighbourhood of residence
Boschi-Pinto et al. (2000) [77] Miyazaki, Japan	965 residents of Village A in Miyazaki Prefecture (389 men, 576 women), followed from 1984 to 1994 with 96% follow-up rate	Anti-HCV, Neg 2 Pos 6	1.0 8.2 (1.6–41.6) Adjusted age, sex, smoking, drinking, and human T lymphotropic virus type I
Mori et al. (2000) [78] Japan	3052 residents of Town K on Kyushu Island (974 men, 2078 women), enrolled in prospective, followed from 1992 to 1997 (mean follow-up: 4.6 years) with 97% follow-up rate	Anti-HCV, Neg 3 Low titre+ 1 High titre+ 18	3.4 (0.35–33.5) 40.4 (11.7–139.2) Adjusted age and sex
Sharp et al. (2003) [79] Japan	7647 members of cohort of Japanese survivors of 1945 atomic bombings who were residents of Hiroshima or Nagasaki and still alive in 1950–1952, autopsied in 1954–1988 with archival tissue samples and clinical records	HCV RNA, Neg 82 Pos 67	1.0 5.9 (2.7–13.4) Adjusted radiation exposure, year of death, age at death, city, sex, HBV infection, and cirrhosis
Wang et al. (2003) [37] Taiwan, China	11,837 men (aged 30–64 years), participants in community-based cancer screening project in 7 townships in main Taiwan Island and Penghu Islets, enrolled between 1990 and 1992 and followed to 2000 (mean follow-up: 7.8 years);	Anti-HCV, Neg 100 Pos 15	1.0 2.45 (1.4–4.2) Adjusted age at recruitment, residence, and HBsAg
Amin et al. (2006) New South Wales, Australia	75,834 HCV monoinfected (without HBV) persons notified to New South Wales Health Department’s Notifiable Diseases Database between 1990 and 2002 (47,903 men, 27,931 women); median age at viral hepatitis notification: 34 years; 10% of cohort estimated to be co-infected with HIV	anti-HCV or HCV RNA, Obs:Exp HCC 143:6.4	22.5 (19.1–26.5) Adjusted age, sex, and calendar year

HCC, Hepatocellular carcinoma; RR, Relative risk

The IARC reviewed 18 case-control studies, after excluding studies with fewer than 20 cases of HCC, studies using controls with chronic epilepsy, and studies where case and controls were difficult to compare for age and sex. Of the 18 studies, 7 were conducted in Europe [60, 63, 65–67, 81, 82], 6 in Asia [48, 50–52, 55, 83], 3 in Africa [70, 72, 84] and 2 in the US [85, 86]. The adjusted odds ratios ranged from 2.8 to 170. In 8 studies, the adjusted odds ratio of HCC exceeded 20 [48, 50, 52, 63, 65, 81, 83, 86]. HBV infection, a potential risk factor for HCC, was investigated in half of all studies, and when the anti-HCV antibody and HCV RNA were identified together, the association was greater than when only anti-HCV antibodies were detected [63, 66, 67, 72].

#### Cholangiocarcinoma

In seven case-control studies of the association between biliary cancer and HBsAg positivity, the odds ratios were positively correlated, ranging from 1.3 to 8.9, and 3 of these studies were statistically significant [87–89]. However, according to IARC assessment, these correlations could have occurred due to the influence of other confounding factors such as HCV infection, clonorchiasis infection, cholelithiasis, alcohol consumption and cirrhosis, and statistical significance was shown only in some studies, so the IARC describes only positive associations in its conclusion.

Studies of the association of HCV with cholangiocarcinoma are rare. In an Australian cohort study, an analysis of the risk of developing cholangiocarcinoma through HCV-only infection did not reveal a statistically significant increase in the standardized incidence ratio. Five case-control studies have been performed in Korea, Japan, and China, 3 of which reported that intrahepatic bile duct carcinoma was associated with HCV positivity [90–92]. In addition, in SEER cancer registry studies, HCV infection was significantly associated with the development of intrahepatic bile duct cancer (adjusted OR, 4.4; 95% CI 1.4–14.0) [93].

#### Non-Hodgkin lymphoma

Totally five cohort studies on the association of HBV infection with non-Hodgkin’s lymphoma were reviewed. Considering the differences in the transmission pathways, only studies conducted in countries with a low prevalence, excluding areas with a high prevalence of HBV infection such as in Asia and Africa, were selected. One study was undertaken in the US [94], another in Australia [44], and the other 3 in Europe [40, 41, 67], respectively. In five cohort studies, three studies analyzed the HIV infection with confounders [44, 67, 94]. In one study not examining HIV infection, the standardized mortality from non-Hodgkin’s lymphoma in HBsAg-positive cases was 3.2 (95% CI 1.2–6.9) and 3.5 (95% CI 1.7–6.2). The relative risk was estimated to be lower than 0.62 to 2.8 when adjusted for HIV infection as a potential confounding variable. In a case-control study, seven of the nine studies showed positive correlations, ranging in size from 1.8 to 4.1.

Compared with HBV, the association between HCV and non-Hodgkin’s lymphoma is consistently high. Of the seven cohort studies investigated (Table 3), five studies examined HIV infection together. In three of these studies, the relative risk of non-Hodgkin’s lymphoma due to HCV infection was two or more and statistically significant [94–96]. One cohort study found statistically significant associations in B-cell non-Hodgkin’s lymphoma [96]. In a cohort study conducted in the US, the attributable risk of non-Hodgkin’s lymphomas to HCV infection was estimated to be approximately 1.28 [97].

**Table 3:** Cohort studies of HCV and lymphoid malignancies

Authors(Years), Country	Cohort description	Detection method, No. of cases	Result RR(95% CI)
Ohsawa et al. (1999) [95] Japan	2162 patients with HCV-related chronic hepatitis (1398 men, 834 women), admitted to 3 medical institutions in Osaka between 1957 and 1997; followed from date of diagnosis to 1997 (average follow-up: 5.7 years)	Anti-HCV, [Obs:Exp] B-cell NHL, 4:1.90	2.1 (0.57–5.4) Adjusted age and calendar year
Rabkin et al. (2002) [117] USA	48,420 gravida, male partners,and offspring from 20,754 pregnancies, recruited from Kaiser Foundation Health Plan (California) between 1959 and 1966	Anti-HCV or HCVRNA, B-cell NHL 0/57 MM 0/24 HD 0/14	No HCV+ controls
Duberg et al. (2005) [96] Sweden	27,150 HCV-infected persons notified to Swedish Institute for Infectious Disease Control between 1990 and 2000 (19,147 men, 8003 women);	Anti-HCV [Obs:Exp] B-cell NHL 20:10.06 CLL 4:2.07 MM 7:2.89	2.0 (1.2–3.1) 1.9 (0.53–4.95) 2.4 (0.97–5.0), Adjusted age, sex, and calendar year
Amin et al. (2006) [44] New South Wales, Australia	75,834 HCV monoinfected (without HBV) persons notified to New South Wales Health Department’s Notifiable Diseases Database between 1990 and 2002 (47,903 men, 27,931 women); median age at viral hepatitis notification: 34 years; 10% of cohort estimated to be co-infected with HIV	[Anti-HCV+ or HCV RNA+], NHL 33 FL 6 Diffuse NHL 19 T-cell NHL, 2 MM 7 HD 4	0.9 (0.6–1.2) 0.6 (0.3–1.4) 1.1 (0.7–1.7) 1.1 (0.3–-4.2) 0.8 (0.4–1.7) 0.4 (0.2–1.1) Adjusted age, sex, and calendar year
UlcickasYood et al. (2007) [94] USA	Cohort of 3888 individuals with chronic HBV infection and comparison cohort of 205,203 individuals without HBV infection;	HCV laboratory test, NHL Neg 116 Pos 3	1.0 2.4 (0.74–7.5), Chronic HBV infection, sex, race and ethnicity, age, Charlson comorbidity score, median household income, and study site
Waters et al. (2005) [118] England	5823 HIV-positive patients at hospital in London, cohort followed since 1996 and seen at regular intervals for clinical assessment; anti-HCV testing introduced in 1998:	Anti-HCV, NHL Neg 56 Pos 7 Not tested 39	1.0 0.97 (0.44–2.1) 1.1 (0.71–1.6)
Franceschi et al. (2006b) [67] Switzerland	Nested case–control study conducted within ongoing cohort study, enrolling people infected with HIV since 1988 from 7 large hospitals in Switzerland; 298 cases of NHL with HCV serology and information on matching criteria occurred through 2004 (246 men, 52 women)	Anti-HCV, NHL Neg 198 Pos 100	1.0 1.05 (0.63–1.75) Adjusted centre, gender, age group, CD4+ count, HIV transmission category, and year of enrollment

RR, Relative risk

Two meta-analyses were performed using 37 case-control studies on HCV infection and non-Hodgkin’s lymphoma [98, 99]. These included only studies in which there were at least 20 cases of lymphoma, studies with no blood lymphoid malignancies in the control group, and studies that could compare case and controls with age and sex. In over 80% of studies, the relative risk of HCV antibody positivity to non-Hodgkin lymphoma or B-cell non-Hodgkin lymphoma was greater than two. These results were observed in all areas except Australia, regardless of the characteristics of the control group. The strength of the association was large, with the odds ratios exceeding five in 13 studies and ranging from 2 to 4 in 15 studies. In addition, the adjusted odds ratio for HCV infection and non-Hodgkin’s lymphoma in the US was found to be 1.35 (95% CI 1.1–1.7, *N* = 33,940) in a US SEER cancer registry study [100]. In pooled analyzes performed on 1465 B-cell lymphomas reported in 5 European countries, the odds ratio was 1.5 (95% CI 0.95–2.2) [101]. Similar results were also seen in a pooled study comparing the seven studies performed in the US, Canada, Australia and Europe (OR, 1.8; 95% CI 1.4–2.3) [102]. The IARC noted that these results indicate that the potential target organ of HCV is a lymphohematopoietic system and it is likely to cause non-Hodgkin lymphoma in the liver and salivary glands. This is consistent with earlier studies [81] that reported similar results. In addition, consistent observations of carcinogenicity in animal and cell experiments support this. Thus, there is sufficient evidence of carcinogenicity due to HCV in humans, especially non-Hodgkin lymphoma.

#### Other cancers

A significant association (OR, 2.3; 95% CI 1.2–4.3) has been found in one epidemiological study of HBV and pancreatic cancer [103], but it was interpreted as resulting from the anatomical sharing of blood vessels and ducts. There was no statistical significance found in studies conducted as part of a Korean cancer prevention study [104]. There has been one case-control study of Hodgkin’s disease with a significant odds ratio (OR, 1.8, 95% CI 0.1–21.5) [105]. One cohort study analyzing the standardized incidence rate for HBV infection and various cancers did not find significant results [44].

Four of the eight case-control studies of HCV and multiple myeloma showed statistically significant results [82, 106–108]. In a large-scale cohort study in the US, HCV infection and acute lymphoblastic leukemia, chronic myelogenous leukemia, and acute non-lymphoid leukemia all showed a positive association, but statistically insignificant [97]. There have been 4 case-control studies that examined the association between HCV infection and leukemia, and no statistical significance was found. In a cohort study conducted in Sweden, Australia and the US, HCV infection and thyroid cancer were analyzed. In Sweden, where five cancers were found, the standardized incidence rate was slightly increased (SIR, 1.55), although this was not significant [96]. In Australia, the risk was reduced (9 cases, SIR, 0.3) [44] and the same result was obtained in the US (46 cases, HR, 0.72; 95% CI 0.52–0.99) [97]. A case-control study was reported in Italy, with statistically significant results (OR, 2.8, 95% CI 1.2–6.3) [82].

### International specific criteria for occupational cancer

Hepatitis viruses are included in the International Labor Organization (ILO) 2015 Occupational Cancer List (revised 2010) as one of nine biological agents [109]. In France, workplace infections with hepatitis viruses A, B, C, D and E are listed on the occupational disease list along with a description of the work involved in exposures [110]. Germany has legally documented the scientific feasibility of occupational diseases in 2014 [111], which is mentioned in the infectious parasitic diseases and tropical diseases section and reports on biological carcinogenesis. The Danish Occupational Disease Inventory of the Carcinogenic or Mutagenic Hazardous Substance Inventory [112] does not include the hepatitis virus. In a review of the CAREX list in Finland [113], each review of HBV and HCV infection suggested that it was difficult to estimate exposure levels, but that the conclusion of the IARC that these infections are highly carcinogenic for HCC should be considered. Exposure data reviewed in the Finnish list reported exposure standards for the ACGIH and 15 other EU member countries in 1995–1996, and no time-weighted average (TWA) or short-term exposure limit (STEL) has been determined yet. The EU Guide for the Diagnosis of Occupational Diseases identifies HBV and HCV as examples in a group of carcinogenic biological hazard factors [114]. However, the relevant literature on the estimation of exposure levels has not been published, so it is impossible to review. In the same guideline, among risk factor exposure level control items, HBV and HCV were not specifically cited. However, the 10 biological carcinogens dealt with in the IARC are discussed and, in accordance with European Directive 2000/54/ EC principles [115], the guidelines emphasize that exposure management is necessary, even if the cancer concerned is nonspecific.

### Consideration issues for specific criteria for occupational cancer

For appropriate specific criteria, the latency period should first be considered. Although there is no scientifically-verified HCC incubation period due to chronic infection with HBV and HCV, it appears that a 20-year period is preferred in the literature after examining epidemiological studies [2, 13, 116]. In addition, most HCCs follow liver cirrhosis and progress to cancer, so the latency period may be up to 40 years [116]. Second, existing studies have defined a serologic diagnosis of HBsAg positive and HCV antibody positive as constituting the exposed group. However, the IARC review identifies chronic infection with HBV or HCV as carcinogenic, and so distinguishes between simple infection and chronic infection. Therefore, rather than diagnosing exposure only through positive antibodies in the serum, it should be assumed for determining specific criteria of occupational cancer that the chronic infection has been clinically confirmed following the infection, after which the clinical course progressed to cancer. Third, consideration of exposure levels and exposure frequency of hepatitis virus infection is necessary. However, as far as can be determined in the literature review, there is no validated consensus concerning exposure levels or on the frequency of virus exposure that may cause HCC. Finally, there is a difference in the carcinogenicity of HBV and HCV, especially in cancers other than HCC. Chronic infection due to HCV is at a level sufficient to induce non-Hodgkin lymphoma, but chronic infection due to HBV has a positive association only with non-Hodgkin’s lymphoma.

## Conclusions

We recommend keeping the current specific criteria "liver cancer caused through exposure to contagious blood". In establishing more detailed occupational cancer specific criteria, the following guidelines are recommended:

Hepatocellular carcinoma: This cancer concerns workers possibly exposed to HBV or HCV-positive blood following injury with a sharp medical device during work and where HBV or HCV infections are confirmed using serologic testing. If infection persists, and the worker is diagnosed as having a chronic infection, and if an HCC is confirmed, it can be considered highly related to the worker’s occupation.

Non-Hodgkin’s lymphoma: This cancer concerns workers possibly exposed to HCV-positive blood following injury with a sharp medical device during work and where HCV infection is confirmed using serological testing. If infection persists, and the worker is diagnosed as having a chronic infection, and if Non-Hodgkin’s lymphoma is confirmed, then it can be considered highly related to the worker’s occupation.
